# The Manchester Meeting

**Published:** 1911-07-01

**Authors:** 


### THE MANCHESTER MEETING.

Hospital representatives from the Manchester and Liverpool districts met at Manchester on Tuesday afternoon to consider the position of hospitals under the Insurance Bill.

Sir William Cobbett, Chairman of the Board of Management of the Manchester Royal Infirmary, presided, and said Mr. Lloyd George had expressed the opinion that hospitals would not be affected by the measure, but the practically unanimous opinion of hospital governors and managers was that such institutions would suffer to an injurious and a very serious degree unless certain alterations were made. Workmen who would benefit from the insurance scheme were substantial and sometimes the most important contributors to hospitals, and in all cases their assistance, joined to that of employers, was very considerable. By interfering with these aids at the source the Bill must hit very hardly the workshop and factory contributions to hospital and infirmary funds both from operatives and employers. Sir F. Forbes Adam proposed that a memorial be presented to Mr. Lloyd George in favour of compensating hospitals for the expenses which they may incur in the treatment of persons voluntarily or compulsorily insured under the Bill. Alderman Park, of Preston, seconded, and remarked that the Preston Infirmary, which was largely dependent upon the contributions of workpeople, would probably lose £2,000 a year if the Bill passed in its present form. The resolution was carried unanimously. We hope to deal at length with the meeting in our next issue.

